# Deletion of RasGRF1 Attenuated Interstitial Fibrosis in Streptozotocin-Induced Diabetic Cardiomyopathy in Mice through Affecting Inflammation and Oxidative Stress

**DOI:** 10.3390/ijms19103094

**Published:** 2018-10-10

**Authors:** Tzu-Hsien Tsai, Cheng-Jei Lin, Sarah Chua, Sheng-Ying Chung, Shyh-Ming Chen, Chien-Ho Lee, Chi-Ling Hang

**Affiliations:** 1 Division of Cardiology, Department of Internal Medicine, Kaohsiung Chang Gung Memorial Hospital and Chang Gung University College of Medicine, Kaohsiung 83301, Taiwan; garytsai@adm.cgmh.org.tw (T.-H.T.); cjlin4@gmail.com (C.-J.L.); chuasr409@hotmail.com (S.C.); miosheny@cgmh.org.tw (S.-Y.C.); syming99@gmail.com (S.-M.C.); gentolata@cgmh.org.tw (C.-H.L.); 2 Institute for Translational Research in Biomedicine, Kaohsiung Chang Gung Memorial Hospital, Kaohsiung 83301, Taiwan

**Keywords:** diabetic cardiomyopathy, RasgRF1, heart failure

## Abstract

Background: Diabetic cardiomyopathy (DCM) is characterized by cardiac fibrosis and stiffness, which often develops into heart failure. This study investigated the role of Ras protein-specific guanine nucleotide releasing factor 1 (RasGRF1) in the development of DCM. Methods: Forty-eight mice were divided into four groups (*n* = 12 per group): Group 1: Wild-type (WT) mice, Group 2: RasGRF1 deficiency (RasGRF1^−/−^) mice. Group 3: Streptozotocin (STZ)-induced diabetic WT mice, Group 4: STZ-induced diabetic RasGRF1^−/−^ mice. Myocardial functions were assessed by cardiac echography. Heart tissues from all of the mice were investigated for cardiac fibrosis, inflammation, and oxidative stress markers. Results: Worse impaired diastolic function with elevation serum interleukin (IL)-6 was found in the diabetic group compared with the non-diabetic groups. Serum IL-6 levels were found to be elevated in the diabetic compared with the non-diabetic groups. However, the diabetic RasGRF1^−/−^ mice exhibited lower serum IL-6 levels and better diastolic function than the diabetic WT mice. The diabetic RasGRF1^−/−^ mice were associated with reduced cardiac inflammation, which was shown by lower invading inflammation cells, lower expression of matrix metalloproteinase 9, and less chemokines compared to the diabetic WT mice. Furthermore, less oxidative stress as well as extracellular matrix deposition leading to a reduction in cardiac fibrosis was also found in the diabetic RasGRF1^−/−^ mice compared with the diabetic WT mice. Conclusion: The deletion of RasGRF1 attenuated myocardial fibrosis and improved cardiac function in diabetic mice through inhibiting inflammation and oxidative stress.

## 1. Introduction

Multiple epidemiologic studies have reported a strong association between heart failure and diabetes mellitus (DM) [1,2,3]. The term diabetic cardiomyopathy (DCM) is used for patients who have heart failure in the absence of other co-morbidities such as coronary artery disease, hypertension, and myocardial infarction [4,5,6]. DCM includes diabetic patients with diastolic heart failure, the prevalence of which is as high as 60% in patients with Type II DM [4,5,6]. The early phase of DCM is characterized by symptoms of diastolic heart failure, which is caused by myocardial fibrosis via excess fibrotic processes, even without the loss of cardiomyocytes (with preserved left ventricular systolic function) [7,8,9]. Myocardial fibrosis is a hallmark of heart disease, and is defined as an abnormal deposition of collagen, fibronectin, and other extracellular matrices, which leads to increased myocardial stiffness and consequent cardiac dysfunction, ultimately resulting in heart failure [10,11,12,13]. Streptozotocin (STZ)-induced diabetic mice with impaired diastolic function, heart failure, and exhibiting features of cardiac hypertrophy, myofibril depletion, and interstitial fibrosis were developed, and are used to study the mechanism of DCM [6,7,14,15]. Therefore, a mouse model of STZ-induced DCM would be a useful and practical mice model for studying the mechanism of DCM. The Ras protein-specific guanine nucleotide releasing factor 1 (RasGRF1) is a family of Ras-selective guanyl exchange factors. The RasGRF1 mediates the activation of inflammation and oxidative stress by regulation of the proteins of the Ras family [16]. In the rheumatoid arthritis, RasGRF1 has been found to contribute to the production of matrix metalloproteinases by regulating inflammation processes [17]. Chronic activation of the Ras family is thought to contribute to diabetes-related complications such as diabetic nephropathy and retinopathy [18,19,20,21]. Additionally, RasGRF1 regulates the activity of the Ras family in aging-related cardiac fibrosis [22]. However, the role of RasGRF1 in DCM induced by chronic diabetes is unclear. Therefore, this study investigated the role of RasGRF1 in heart failure induced by chronic diabetes.

## 2. Results

### 2.1. Establishment of Diabetic Mouse Model

**Figure 1:**
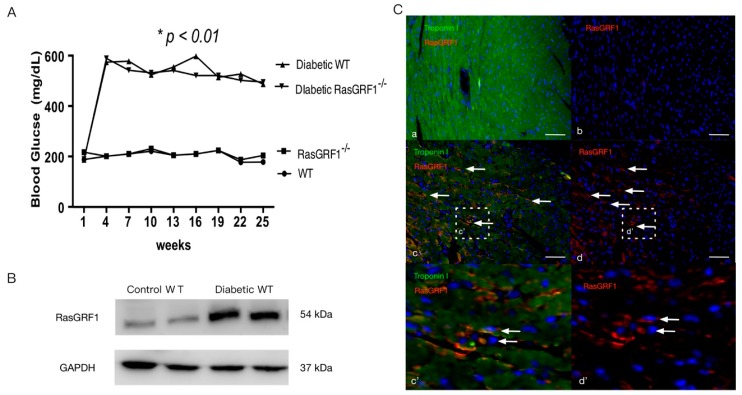
Dynamic expression of Ras protein-specific guanine nucleotide releasing factor 1 (RaGRF1) in the cardiac tissue of non-diabetic and diabetic mice. (**A**) Peripheral blood glucose concentration was measured once every two weeks. Fasting blood sugar level was measured biweekly in wild-type (WT) B6 mice, RasGRF1^−/−^ mice, diabetic WT B6 mice, and diabetic RasGRF1^−/−^ mice (each group, *n* = 12). Values represent mean ± SEM (*n* = 12). The diabetic mice exhibited higher fasting blood sugar levels than the sham control, but there was no difference between diabetic WT B6 mice and diabetic RasGRF1 deficiency (RasGRF1^−/−^) mice. * *p* < 0.01, vs. non-diabetic groups; (**B**) Mice were divided into sham control (*n* = 2) or 24-week period diabetic wild-type (WT) mice (*n* = 2) induced by streptozotocin (STZ) injection. Immunoblotting for RasGRF1 was performed, and it was observed that the expression of RasGRF1 was significantly upregulated in diabetic wild-type mice compared to control WT mice. (**C**) Dual immunofluorescence staining was performed with RasGRF1 (red) and troponin I (green) in sham control and diabetic WT mice. An increased expression of RasGRF1 (white arrow) in diabetic mice (a–d) was observed and the positive RasGRF1 cells were not co-localized with troponin I. (c’ and d’ are enlarged images of c and d) WT: Wild-type.

Streptozotocin (STZ) (50 mg/kg body weight) was injected consecutively for five days to induce diabetes mellitus in WT (wild-type) B6 and RasGRF1 deficiency (RasGRF1^−/−^) mice. Two weeks after injection, 85% of total mice exhibited fasting blood glucose levels higher than 350 mg/dL. These mice were considered diabetic mice, and were used for further experiments. The administration of STZ was toxic to approximately 5% of the mice, whereas 10% of mice did not develop diabetes. Only the surviving diabetic mice (*n* = 12 each in group 3: diabetic WT mice and group 4: diabetic RasGRF1^−/−^ mice) with fasting blood glucose levels of >350 mg/dL throughout the study period were used for this study. As shown in Figure 1A, no difference in blood glucose levels between diabetic WT mice and diabetic RasGRF1^−/−^ mice were observed throughout the study period. However, the blood sugar levels were significantly higher in diabetic mice (groups 3 and 4) than in the non-diabetic groups (group 1: wild-type (WT) mice, group 2: RasGRF1^−/−^ mice).

### 2.2. The Express of RasGRF1 Is Increased in Cardiac Fibroblasts of Diabetic Mice

To determine whether diabetic condition induced the upregulation of RasGRF1, immunoblotting was performed to compare the RasGRF1 level between diabetic and non-diabetic B6 mice. The level of RasGRF1 was significantly upregulated in the heart of diabetic wild-type mice compared with non-diabetic mice at 24 weeks (Figure 1B). Dual IF (immunofluorescence) staining with RasGRF1 and troponin I demonstrated the upregulation of RasGRF1 in the cardiac fibroblasts of diabetic mice.

### 2.3. RasGRF1 Deficiency Mice Lowered the Circulation IL-6 Level and Improved Diastolic Function in Diabetic Mice

**Figure 2:**
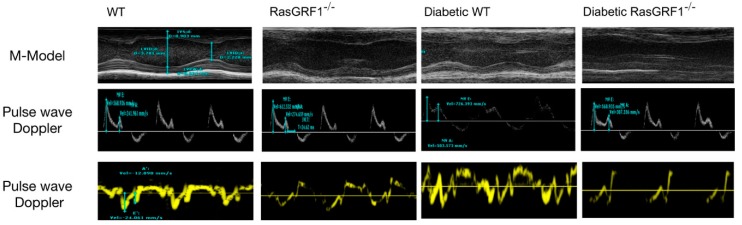
Morphology and cardiac function in mice as measured by cardiac echography. Representative echocardiography (**upper panel**) showed M-mode tracing. Lines indicate left ventricular septum thickness in diastole (LVSd), left ventricular posterior wall thickness in diastole (LVPWd), left ventricular internal dimension in diastole (LVIDd), left ventricular internal dimension in systole (LVIDs), and left ventricle (LV) mass. Pulse wave doppler recordings of mitral valve leaflet tips (**middle panel**) showed mitral inflow velocity patterns, and were also calculated. Representative tracings (**lower panel**) for tissue Doppler imaging of the mitral annulus septum. The E′ wave indicated the motion of the mitral annulus during early diastolic filling of the LV, and A′ wave indicates the atrial systole during late filling of the LV. LV: left ventricle.

The diabetic WT mice group showed higher interleukin (IL)-6 levels than the non-diabetic groups (group 1 and group 2). However, the diabetic RasGRF1^−/−^ mice had lower IL-6 levels than the diabetic WT mice group (Table 1). Cardiac echocardiography was performed at 24 weeks after the mice were diagnosed as diabetics. As shown in Table 1 and Figure 2, diabetic WT mice displayed cardiac dysfunction, as indicated by the slight reduction of both the ejection fraction (EF) and fractional shortening (FS), as compared to the control groups. However, the ratios of the reduced early and late diastolic flow velocity (E/A) and early diastolic flow velocity versus the mitral annulus early diastolic velocity (E/E’) were found in the diabetic WT mice compared with the control groups, indicating abnormality in the left ventricular diastolic function. It was observed that a chronic diabetic condition induced left ventricular ejection fraction and preserved heart failure (with adequate systolic function and abnormal diastolic function. Interestingly, such changes were prevented in diabetic RasGRF1^−/−^ mice (Table 1).

### 2.4. Deficiency of RasGRF1 Attenuated Cardiac Fibrosis in Diabetic Mice

**Figure 3:**
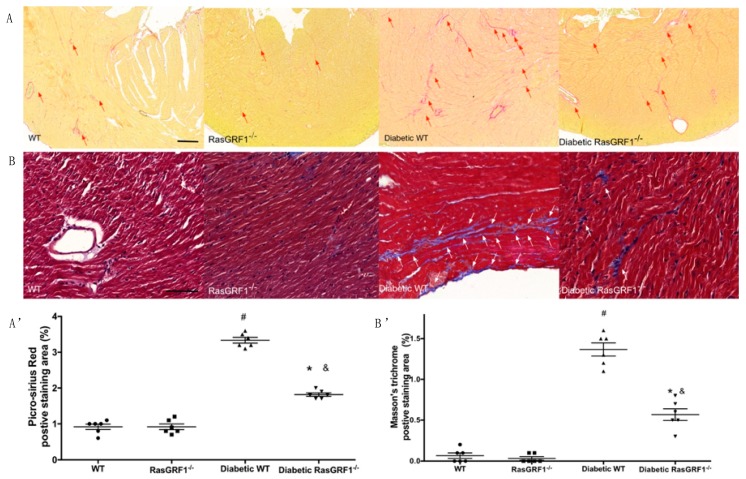
Effect of RasGRF1 knockout on cardiac fibrosis in diabetic mice. The deposition of collagen in cardiac tissue by picrosirius red staining (**A**) and Masson’s trichrome staining (**B**). (**A**,**A’**) The picrosirius red staining was in different groups (each group *n* = 3). The red arrow indicated interstitial fibrosis and the red arrow indicated the perivascular fibrosis. ^#^ *p* < 0.01 vs. normal WT and normal RasGRF1^−/−^ mice; * *p* < 0.05 vs. diabetic WT mice. ^&^ *p* < 0.05 vs. normal WT and normal RasGRF1^−/−^ mice. (**B**,**B’**) The Masson’s trichrome staining in different groups (each group *n* = 3). The white arrow indicated fibrotic area. ^#^ *p* < 0.01 vs. normal WT and normal RasGRF1^−/−^ mice; * *p* < 0.05 vs. diabetic WT mice. ^&^ *p* < 0.05 vs. normal WT and normal RasGRF1^−/−^ mice. WT: wild type; Scale bar = 200 μm.

Picrosirius red and Masson’s trichrome staining were performed to detect whether diabetes mellitus induced cardiac fibrosis. Collagen deposition as measured by Picrosirius red stain was significantly increased in diabetic WT mice compared to WT and RasGRF1^−/−^ groups (group 1 and groups 2) (Figure 3A). This increase was significantly reduced in diabetic RasGRF1^−/−^ mice compared to diabetic WT mice. Focal scarring, which was observed using Masson’s trichrome stain, was higher in diabetic WT mice than the sham control groups, and this change was prevented in RasGRF1^−/−^ mice (Figure 3B).

### 2.5. Deficiency of RasGRF1 Prevented Inflammatory Cytokine and Extra-Matrix Deposition Production in the Hearts of Diabetic Mice

**Figure 4:**
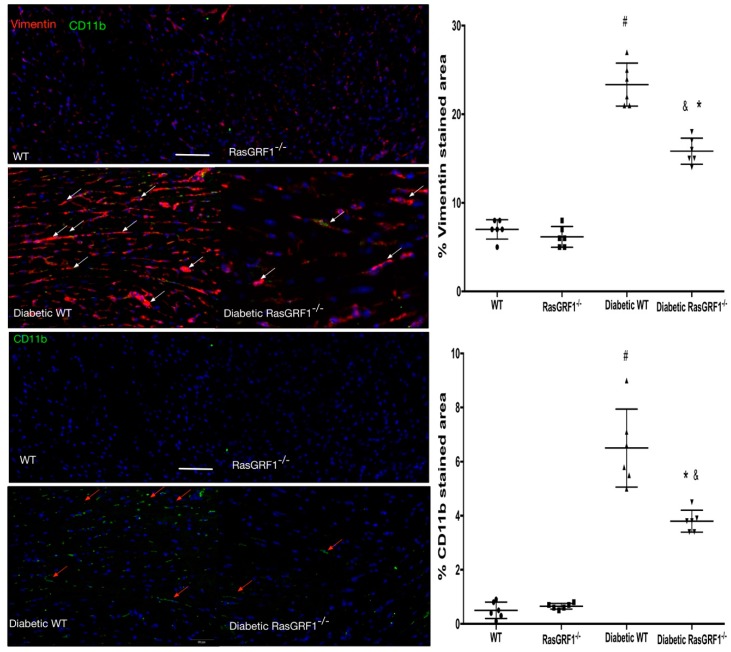
Effects of RasGRF1 knockout on the expression of CD11b and Vimentin in diabetic mice. Tissue sections were stained with double immunofluorescence staining for vimentin (red) and CD11b (green) expressing cells. Sham control mice (all *n* = 4 in WT and RasGRF1^−/−^ mice; no difference between these two groups was observed); Diabetic WT and diabetic RasGRF1^−/−^ mice (all *n* = 4 in each group); the white arrow indicated a vimentin-positive area, and the red arrow indicated the CD11b positive cells. In contrast to diabetic WT mice, diabetic RasGRF1^−/−^ mice in an attenuated chronic diabetic condition induced monocytes infiltration and vimentin deposition. ^#^ indicate *p* < 0.01 vs. normal WT and normal RasGRF1^−/−^ mice; * *p* < 0.05 vs. diabetic WT mice. ^&^ *p* < 0.05 vs. normal WT and normal RasGRF1^−/−^ mice. Scale bar = 200 μm.

**Figure 5:**
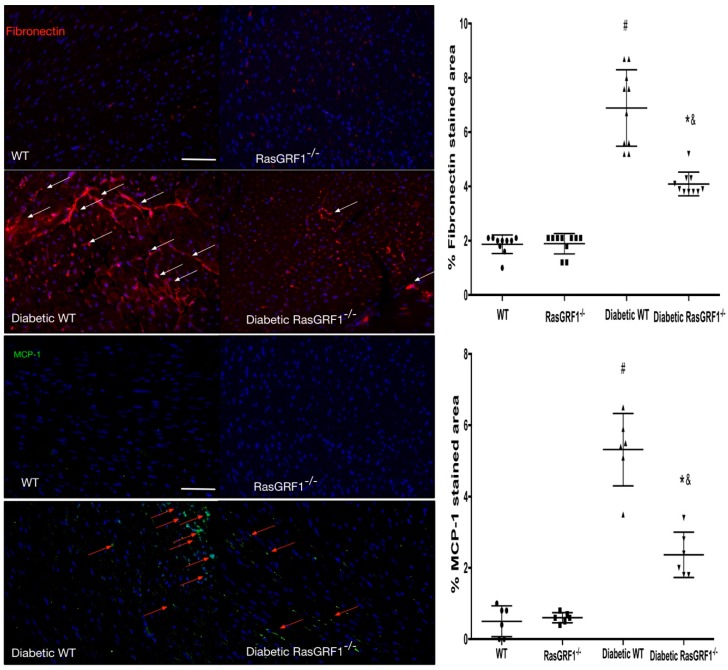
Effects of RasGRF1 knockout on the expression monocyte chemoattractant protein-1 (MCP-1) and fibronectin in diabetic mice. Tissue sections were stained with dual immunofluorescence staining for MCP-1 expressing and fibronectin expression cells. The control mice received saline (*n* = 4 WT, *n* = 4 RasGRF1^−/−^; no difference between these two groups was observed); diabetic WT and RasGRF1^−/−^ mice (each group *n* = 4). The white arrow indicated a fibronectin positive area, and the red arrow indicated the MCP-1 positive cells. Diabetic RasGRF1^−/−^ mice in an attenuated chronic diabetic condition induced interstitial collagen deposition and the expression of pro-inflammatory cytokines compared to diabetic WT mice. ^#^ *p* < 0.01 vs. normal WT and normal RasGRF1^−/−^ mice; * *p* < 0.05 vs. diabetic WT mice. ^&^ *p* < 0.05 vs. normal WT and normal RasGRF1^−/−^ mice. Scale bar = 200 μm.

Monocytes have major roles in initiating and promoting inflammatory processes, which are a major cause of cardiac fibrosis. We observed higher positive CD11b and vimentin cells in diabetic mice (group 3 and group 4) than in the non-diabetes group (group 1 and 2) (Figure 4). These findings were reversed in the diabetes-RasGRF1^−/−^ group. The monocyte chemoattractant protein-1 (MCP-1) is one of the chemokines that regulates the migration of monocytes, whereas fibronectin is a well-known extracellular matrix protein. The diabetes WT mice showed significant upregulation of MCP-1 and fibronectin-positive cells compared to the control group and the RasGRF1^−/−^ group (Figure 5). In addition, the diabetic RasGRF1^−/−^ mice attenuated the expression of MCP-1 and reduced the fibronectin deposition.

### 2.6. Effects of Deficiency of RasGRF1 on the mRNA Expression of Inflammatory Cytokines, Oxidative Stress, and Extracellular Matrix in Diabetic Mice

**Figure 6:**
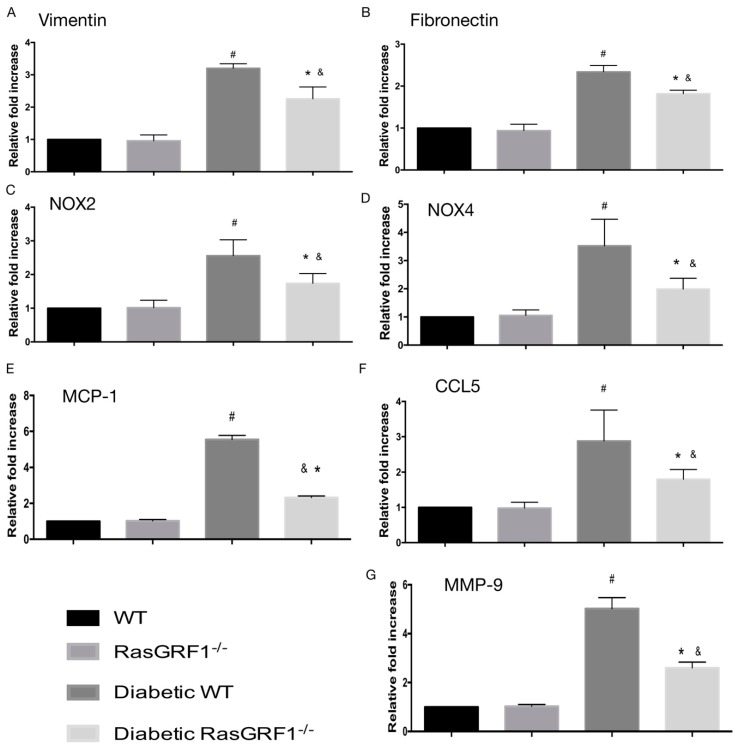
Effects of RasGRF1 knockout on mRNA expression in diabetes-induced extracellular matrix accumulation, inflammation cytokines and oxidative stress. The diabetic condition caused the upregulation of the extracellular matrices of vimentin, fibronectin, oxidative stress, NOX2 and NOX4, and the inflammatory cytokines and chemokines mRNA, MCP-1, chemokine (C–C motif) ligand 5 (CCL5), and matrix metalloproteinase 9 (MMP-9) levels of heart tissue (*n* = 5). In additional, the diabetic condition-induced upregulation of the extracellular matrices, oxidative stress, inflammatory cytokines and chemokines, and mRNA can be inhibited by RasGRF1^−/−^ mice. Data are shown as mean ± SEM from three independent experiments. ^#^ *p* < 0.01 vs. normal WT and normal RasGRF1^−/−^ mice; * *p* < 0.05 vs. diabetic WT mice. ^&^ *p* < 0.05 vs. normal WT and normal RasGRF1^−/−^ mice. WT: wild-type.

Figure 6 shows that the upregulation of these inflammatory chemokines and cytokines such as MCP-1, chemokine (C–C motif) ligand 5 (CCL5), and matrix metalloproteinase 9 (MMP-9) levels was significantly higher in diabetic WT mice (group 3) than in the non-diabetic groups (groups 1 and 2). However, mRNA expression of these inflammatory chemokines and cytokines were attenuated in diabetic RasGRF1^−/−^ mice (group 4) compared to diabetic WT mice (group 3). In addition, the diabetic WT mice (group 3) also significantly increased the mRNA expressions of NOX2, NOX4, vimentin, and fibronectin over the non-diabetic groups (groups 1 and 2). In the diabetic RasGRF1^−/−^ mice, these gene expressions were reversed.

### 2.7. Effects of Deficiency of RasGRF1 on Protein Expressions of Oxidative Stress and Extracellular Matrix in Diabetic Mice

**Figure 7:**
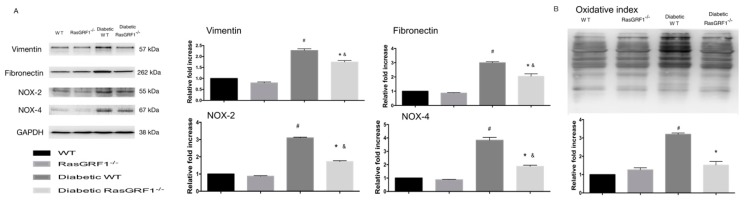
Effects of Rasgrf1 knockout prevented diabetes-induced oxidative stress and extramatrix accumulation (**A**) Chronic diabetes upregulated the proteins of the extracellular matrix markers vimentin and fibronectin, and oxidative stress markers NOX2 and NOX4 in heart tissue. However, the upregulation of these proteins was attenuated in diabetic RasGRF1^−/−^ mice (*n* = 3). The protein levels are expressed as a ratio to β-actin, and normalized to sham control wild-type (WT). (**B**) The oxidative index and protein carbonyls were substantially higher in diabetic WT mice than in the control groups and diabetic RasGRF1^−/−^ mice (*n* = 3). Data are shown as mean ± SEM in three independent experiments. ^#^ *p* < 0.01 vs. normal WT and normal RasGRF1^−/−^ mice; * *p* < 0.05 vs. diabetic WT mice. ^&^ *p* < 0.05 vs. normal WT and normal RasGRF1^−/−^ mice. WT: wild-type.

Figure 7 shows that diabetic WT mice (group 3) had a significantly higher regulation of oxidative proteins of NOX2 and NOX4 compared to the non-diabetes groups (groups 1 and 2). However, the expression of these proteins was significantly lower in diabetic RasGRF1^−/−^ mice. In additional, the diabetic WT mice revealed an upregulated expression of vimentin and fibronectin. The RasGRF1^−/−^ mice significantly reduced the expression of these proteins compared to the diabetic WT mice. The pattern of expression of oxyblot heart proteins were also attenuated in diabetic RasGRF1 mice.

### 2.8. Effects of Deficiency of RasGRF1 on Protein Expressions of Inflammatory Cytokine and Chemo-Attractive Cytokines in Diabetic Mice

**Figure 8:**
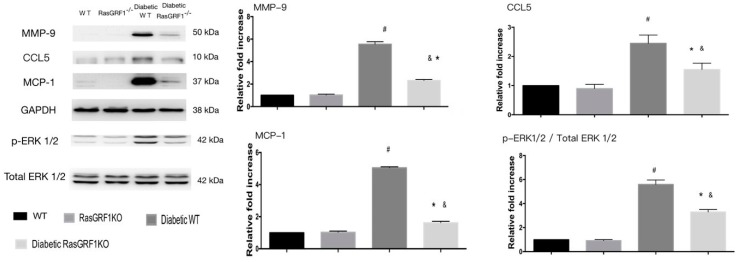
Effects of Rasgrf1 knockout prevented diabetes-induced inflammation processes through the ERK pathway. Chronic diabetes status upregulated the proteins of inflammatory cytokines and chemokines makers: including monocyte chemoattractant protein-1 (MCP-1), chemokine (C–C motif) ligand 5 (CCL5), and MMP-9: (*n* = 3). These upregulation proteins were prevented in RasGRF1^−/−^ mice. The protein levels are expressed as a ratio to β-actin, and normalized to sham control wild-type (WT). In additional, the chronic diabetic status induced the *phosphorylation of* ERK1/2. The diabetic RasGRF1-attenuated diabetic status induced the *phosphorylation of* ERK1/2. The protein levels are expressed as a ratio to total ERK1/2 and normalized to sham control wild-type (WT). Data are shown as mean ± SEM in three independent experiments. ^#^ *p* < 0.01 vs. normal WT and normal RasGRF1^−/−^ mice; * *p* < 0.05 vs. diabetic WT mice. ^&^ *p* < 0.05 vs. normal WT and normal RasGRF1^−/−^ mice. WT: wild type.

To check whether a decrease in the mRNA expressions of inflammatory cytokines and chemokines were reflected in the protein expression, Western blot of MCP-1, CCl5, and MMP-9 was carried out. Figure 8 shows the upregulation of chemoattractive and inflammatory cytokines of MCP-1, CCL5, and MMP-9 were significantly higher in diabetic WT mice (group 3) compared to the non-diabetes group (group 1 and group 2). However, the expression of these proteins was lower in diabetic RasGRF1^−/−^ mice compared to diabetic WT mice. In addition, the expression of these proteins were increased by the phosphorylation of ERK1/2 in diabetic WT mice. In contrast, RasGRF1^−/−^ mice significantly reduced ERK1/2 phosphorylation. Collectively, these data strongly suggested that long-term diabetes conditions upregulated MCP-1, MMP-9, NOX2, and NOX4 via ERK2/3 phosphorylation.

## 3. Discussion

This study demonstrates that a long-term diabetes condition can upregulate the RasGRF1 expression in heart tissue. This study revealed a novel role for RasGRF1 signaling in DCM. The chronic diabetes condition increased the expressions of RasGRF1 in cardiac fibroblast. Phosphorylation ERK signaling increased the expression of pro-inflammatory cytokines (CCL5 and MCP-1). This in turn triggered monocyte infiltration to the myocardium, which then caused inflammation and oxidative stress processes. These processes induced the cardiac fibroblasts to produce an abundant extracellular matrix in myocardium. Deposition of the abundant extracellular matrix in the myocardium then caused diastolic heart failure in diabetic mice. A deficiency of RasGRF1 attenuated these processes and improved diastolic function in diabetic mice.

### 3.1. Inflammatory Process and Oxidative Stress Are Major Causes of DCM

Patients with diabetes exhibit chronic low-grade inflammation [23,24,25,26,27,28]. The diabetic cardiomyopathy is characterized by diastolic dysfunction in humans, and is caused by myocardial stiffness by interstitial and perivascular fibrosis, which themselves are caused by reactive fibrotic processes, even without the loss of cardiomyocytes (with preserved left ventricular systolic function) [7,8,9]. Hyperglycemia-induced inflammatory and oxidative stress can cause substaintial transcriptional changes in the cells, resulting in an augmented production of cytokines, including several inflammatory mediators (MCP-1 and CCL5). In DCM, these mediators induced infiltration of the inflammatory cells into the myocardium, where they secrete inflammatory cytokines through the phosphorylation of ERK2/3 pathways in diabetic cardiomyopathy [23,24,29]. Our results are consistent with previous findings [23,24,29] and demonstrate that DM induces chronic inflammation that can cause substantial functional and structural alterations in the heart tissue.

### 3.2. RasGRF1-Regulated Inflammatory Cytokines Secrete an Endoplasmic Reticulum-Dependent Reactive Oxidant Species Production

The Ras superfamily of small GTPases are expressed in mammalian tissue, have a pivotal role in the response to extracellular stimuli, and activate multiple downstream proliferation signaling pathways [30]. A recent study has found that RasGRF1 in synovial fibroblasts regulates the inflammatory process by producing matrix metalloproteinases (MMPs) that destroy the joint structure in rheumatoid arthritis [17]. The abnormal expression of RasGRF1 also participates in the aging-induced secretion of inflammatory cytokines by cardiac fibroblast. This in turn promoted monocyte transition into myeloid fibroblasts, resulting in cardiac fibrosis in the aging mouse heart. The inhibition of RasGRF1 activity by drugs was effective in attenuating aging-induced cardiac fibrosis and heart failure [22]. In addition, the RasGRF1 also participated in reactive oxidant species production with endoplasmic reticulum (ER) stress [16]. Although it is known that inflammatory processes and oxidant stress via ER are still the major mechanisms of diabetic cardiomyopathy [7,8,9], it is still unclear whether RasGRF1 also regulates the long-term diabetic condition-induced cardiac fibrosis. Our study was performed to clarify this concept.

Diabetic condition triggers the upregulated expression of RasGRF1 in heart tissue in the present study. Here, we demonstrated the upregulation expression of RasGRF1 in the heart tissue of diabetic mice. The major source of the upregulation of RasGRF1 is cardiac fibroblast in a diabetic heart. Previous studies had found that fibroblasts are acquired as an inflammatory phenotype, which is characterized with the release of MCP-1, IL-6, and IL-8 [22,31]. These inflammatory fibroblasts can drive the circulating monocyte via the cytokine-induced expression of adhesion molecules. These monocytes may secrete inflammatory cytokines to induce fibroblasts to release pro-inflammatory cytokines to degrade the extracellular matrix and increase fibroblast proliferation and collagen production. Our findings also identified that the diabetic condition increased the chemokines (MCP-1 and CCL5) to attract the monocyte to infiltrate into the myocardium, which secretes pro-inflammatory cytokines (MMP-9) and promotes the deposition of the extracellular matrix. These findings supported the previously reported data and highlighted the importance of inflammation processes in cardiac fibrosis as previously reported [22,31].

### 3.3. The Role of RasGRF1 in DCM and Clinical Impact

**Figure 9:**
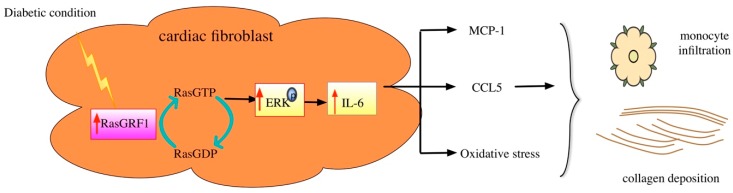
The possible role of RasGRF1 in diabetic cardiomyopathy.

Our study revealed that diabetic RasGRF1^−/−^ mice were with lower serum IL-6 levels compared to diabetic WT mice. The diabetic RasGRF1^−/−^ mice showed a significant reduction in the serum levels of IL-6, as well as monocyte infiltration via a decreased secretion of MCP-1 and CCL5. Our study and previous study also demonstrated an elevation of serum IL-6 levels in diabetic mice [32]. The treatment of IL-6 alone promoted collagen production in cultured cardiac fibroblasts and the interstitial fibrosis of the rat heart [32]. Remarkably, the deletion of IL-6 was also shown to be beneficial against diabetic cardiomyopathy through the attenuation of the cardiac fibrosis process [27]. The pro-inflammatory cytokines, oxidative stress, and extracellular matrix were also sequentially diminished to attenuate cardiac fibrosis in diabetic RasGRF1^−/−^ mice compared to WT. Consistent with our data, the increased expression of RasGRF1 also has been found in rheumatoid arthritis and aging-related cardiac fibrosis [17,22]. The other important finding of our study is that the RasGRF1 regulates the downstream cellular signal pathway through the phosphorylation of the ERK pathway. In our report, we demonstrated that the diabetic cardiac fibroblasts had a marked increase of RasGRF1, which is responsible for the augmented IL-6 synthesis and increase inflammation and oxidative stress which results in cardiac fibrosis in diabetic mice (Figure 9). Collecting the above findings, we found RasGRF1 to be an important therapeutic target in the prevention and treatment of diabetic cardiomyopathy.

The diabetic condition increased the expression of RasGRF1 in cardiac fibroblasts, which promoted the exchange of GDP for GTP on Ras and led to Ras activation. This activation induced the phosphorylation of ERK, which in turn induced IL-6 expression. Finally, IL-6 augmented the expression of key inflammatory and oxidative stress markers, resulting in inflammatory cells infiltration and collagen deposition in diabetic mice. This collectively promoted cardiac fibrosis.

Statin is known to reduce cholesterol synthesis through the inhibition of the mevalonate pathway [33]. Statin is included in major classes of drugs, which have been found to block protein farnesylation by inhibiting the production of farnesyl pyrophosphate [33]. These showed the ability of statin to inhibit cell proliferation by blocking protein prenylation [33,34]. This potential clinical use of statin for inhibiting protein prenylation has raised interest in studying its effect on inhibiting the growth of cardiac fibroblasts. Since RasGRF1 activation can be controlled by Farnesyl transferase (FTase) activity, statins are effective for inhibiting FTase activity through the inhibition of farnesyl pyrophosphate production [22,34]. Interestingly, statin is used to block the activation of RasGRF1 in daily clinical practice [35]. Our study indicates that statins can be used to prevent DCM in patients with DM. Furthermore, recent studies also indicated statins to be effective for attenuating DCM [36,37]. The results of our study provide further evidence that statin can reduce the cardiovascular complications in patients with DM.

This study has several noticeable limitations. First, the experimental mice that were used in this study had blood sugar levels of 400–600 mg/dL, and the duration of diabetes was of six months. Although the diabetic period in our study was longer than the previous studies, no left ventricular-reduced ejection fraction-mediated heart failure was observed in our mice. This finding differs from previous studies and may be attributed to the use of different strains of mice between the studies. Second, we did not directly define the pivotal role of RasGRF1 in the development of cardiac fibrosis through the use of cardiac fibroblast specific RasGRF1 knockout mouse, since it is currently unavailable. Third, long-term uncontrolled diabetes induced significant impaired diastolic dysfunction in mice, but some differences can arise between clinical and experimental conditions. Moreover, the STZ-induced diabetic mice model was used in our experiment. We cannot exclude the possibility of the direct cardiac toxicity of STZ. However, the STZ-induced DCM is widely used in experimental studies. Overall, these reasons are of concern for the translation of our findings to clinical practice.

## 4. Materials and Methods

### 4.1. Animals

All of the animal experiments were performed in accordance with the Guide for the Use and Care of Laboratory Animals and approved by the IACUC: Committee (IACUC number: 2016032803 )of Kaohsiung Chang Gung Memorial Hospital, Kaohsiung, Taiwan. The wild-type C57BL/6 mice and C57BL/6N-*Rasgrf1*^tm1aNarl^/Narl (RMRC13257, GEMMS NLAC, Taiwan) mice were purchased from NLAC, Taiwan. The genotypes of the homozygote of RasGRF1^−/−^ mice were confirmed using PCR with specific primers: RasgrfL-forward: AGCAAGTGTCTCACAACTCTGGCAT; Rasgrf1L-Reverse: AAGTTTCAGAACAGCCAAGCTTTGG. 

### 4.2. Diabetic Mice Model Induction and Study Protocol

Mice (*n* = 48) were divided into four experimental groups (*n* = 12/group), Group 1: Wild-type (WT) mice, Group 2: RasGRF1KO mice, Group 3: Diabetic WT mice and Group 4: Diabetic RasGRF1KO mice. Diabetes mellitus was induced in 12-week-old male C57BL/6 or RasGRF1KO mice (body weight of 18–22 g), by intraperitoneal injection of STZ at a dose of 50 mg/kg body weight for five consecutive days. The fasting blood sugar levels were checked once every two weeks using One Touch Ultra (Lifescan, Johnson and Johnson, New Brunswick, NJ, USA) glucometer by tail vein puncture blood sampling. Mice with blood glucose levels of ≥400 mg/dL for three consecutive readings, per week after STZ injection, were considered diabetes.

Cardiac echocardiography was performed on animals under light anesthesia (1% isoflurane) at day 170 after confirming the diabetic condition. Echocardiographic parameters such as heart rate (HR), parasternal long-axis, and short-axis views were recorded by a transthoracic echocardiography system (Vevo 2100, Visual Sonics, Toronto, ON, Canada) equipped with a high-frequency ultrasound probe. The end diastolic thickness of the intraventricular septum, left ventricular (LV) posterior wall thickness (LVPW), and end-systolic LV dimensions were measured on parasternal short-axis M-mode. The fractional shortening (FS), ejection fraction (EF), LV volume, and LV mass index were calculated by Vevo 2100 software (Table 1). All of the data were recorded in triplicate for each mouse. At the end of the six months, after confirmation of the diabetic condition, all of the mice from each group were euthanized for further analysis.

### 4.3. ELISA for Measure of Serum IL-6 Level

Soluble murine IL-6 level in the plasma was detected by ELISA (R&D Systems; Minneapolis, MN, USA; Mouse IL-6 ELISA Ready-SET-Go! Kit) in murine plasma. The protocol was performed as according to the manufacturer’s intructions, and each mouse sample was measured in three experiments described below as A, B, and C.

#### 4.3.1. Histology and Immunofluorescent Staining

The hearts were excised and fixed in 10% formalin, embedded in paraffin, and sectioned at 5-μm thickness. The picrosirius red stain and Masson’s trichrome stain were used to detect collagen content in the cardiac tissues. The positively stained areas of the left ventricular myocardium were examined by using a 100× objective and an average of five fields per mouse was calculated. The index of the positively stained area was calculated as the percentage of positively stained cells per the total area of the left ventricle. 

For immunofluorescent staining, slides were incubated with primary antibodies against fibronectin (Abcam, Cambridge, UK), vimentin (Abcam, Cambridge, UK), CD11b (Biolegend, San Diego, CA, USA), monocyte chemoattractant protein-1 (MCP-1) (Abcam) at 4 °C overnight, and then with a fluorescent dye-conjugated secondary antibody (Invitrogen). Nuclei were visualized by staining with 4′6-diamidino-2-phenylindole dihydrochloride (DAPI) (Santa Cruz Biotechnology, Dallas, TX, USA). The positively stained cells of the left ventricular myocardium were examined by using a 20× objective and an average of five fields per mouse was calculated. The index of positively stained area was calculated as the percentage of positive cells per total number cells. All of the images were captured using immunofluorescent microscope (Olympus BX53, Tokyo, Japan).

#### 4.3.2. Quantitative Realtime qPCR 

Total RNA was extracted using the GENEzol™ TriRNA Pure Kit (Geneaid, New Taipei City, Taiwan) according to the manufacturer’s protocol. cDNA was synthesized from 1 μg of total RNA, and random hexamers by using the Transcriptor First Strand cDNA Synthesis Kit (Roche, Basel, Switzerland). Forward and reverse primers (Table 2) were designed from exon sequences of the target genes to avoid amplification of the genomic DNA. Real time PCR was performed using 1 L of diluted cDNA in a total reaction volume of 20 L using QuantiNova™ SYBR Green PCR kit (QIAGEN, Hilden, Germany) according to manufacturer’s instructions. Real time PCR was performed on Step One Plus Real-Time PCR system (Applied Biosystems, Foster City, CA, USA), with 40 cycles of: 95 °C for 5 s, 60 °C for 1 min. All of the reactions were performed in triplicate, and the data were averaged and analyzed. 

#### 4.3.3. Western Blotting

Cell lysates from cardiac tissue were extracted using RIPA buffer (150 mM of NaCl, 0.1% SDS, 0.5% sodium deoxycholate, 1× protease inhibitor ocktail (Roche, Basel, Switzerland), and 50 mM of Tris; pH 8.0), and the total protein concentration of cell extracts were determined using a BCATM protein assay kit (Pierce, Waltham, MA, USA). Immunoblotting was performed with SDS polyacrylamide gels, and PVDF membrane filter paper. The washing buffer was 0.1% Tween 20 in tris-buffered saline (20 mM Tris and 150 mM of NaCl; pH 7.4), and the blocking buffer was 5% skim milk (Bio-Rad, Hercules, CA, USA) in washing buffer. The following proteins were examined: vimentin (Abcam), fibronectin (Abcam), NOX2 (Millipore, Burlington, MA, USA), NOX4 (Millipore, Burlington, MA, USA), MCP-1 (Abcam), CCL5 (GeneTex, Irvine, CA, USA), MMP9 (GeneTex), total ERK1/2 (Cell Signal, Danvers, MA, USA), and phospholyration-ERK1/2 (Cell Signal). The Oxyblot Detection kit (Millipore) was used and modified as previously reported [38] to determine the detect levels of heart-oxidized proteins from total proteins.

## 5. Statistics

All of the data were presented as mean ± SEM. Statistical analyses were performed using SPSS 18.0 (SPSS Inc., Chicago, IL, USA). Data were analyzed using the Student t-test or one way ANOVA, followed by a Bonferroni multiple-comparison post hoc test. *P* < 0.05 was considered to be statistically significant.

## 6. Conclusions

The regulation of RasGRF1 attenuates myocardial fibrosis and improves cardiac function by inhibiting inflammation and oxidative stress in diabetic mice.

## Figures and Tables

**Table 1:** Body weight, heart weight, and serum interleukin (IL)-6 levels and cardiac performance parameters measured by echocardiography.

	Control (WT B6)	RasGRF1^−/−^	Diabetes WT	Diabetes RasGRF1^−/−^
Blood glucose (mg/mL)	172 ± 16	162 ± 12	542 ± 43 *	587 ± 52 *
BW (g)	32.2 ± 0.48	29.5 ± 0.54 ^&^	25.8 ± 0.42 *	26.1 ± 0.36 *
HW (mg)	162.2 ± 3.2	157.2 ± 3.2	138.9 ± 4.3	134.2 ± 4.1
HW/BW	4.67 ± 0.11	4.42 ± 0.13	4.51 ± 0.18	4.78 ± 0.17
IL-6 (ng/mL)	234 ± 21	198 ± 19	764 ± 78 *	674 ± 56 ^&^
LVSd (mm)	0.78 ± 0.05	0.78 ± 0.06	0.71 ± 0.03	0.72 ± 0.05
LVPWd (mm)	0.92 ± 0.06	0.89 ± 0.07	0.87 ± 0.06	0.89 ± 0.05
LVIDd (mm)	2.63 ± 0.31	2.58 ± 0.17	3.12 ± 0.21 *	2.89 ± 0.13 *
LVIDs (mm)	1.42 ± 0.13	1.52 ± 0.18	1.98 ± 0.19 *	1.73 ± 0.13 *
LV mass index	101 ± 21	98 ± 18	86 ± 32 *	88± 29 *
EF (%)	67 ± 8.1	68 ± 7.2	51 ± 6.3 *	54 ± 7.4*
FS	45 ± 6.3	43 ± 5.4	37 ± 6.2 *	39 ± 7.2 *
E/E′	17 ± 3.8	16 ± 4.2	27 ± 3.8 *	21 ± 3.4 ^&^
E/A	1.6 ± 0.76	1.5 ± 0.81	1.1 ± 0.89 *	1.3 ± 0.76 ^&^

LVSd, diastolic interventricular septum diameter; LVIDd, diastolic le ventricular internal diameter; LVIDs, systolic le ventricular internal diameter; LVPWd, diastolic left ventricular posterior wall diameter, EF: ejection fraction, FS: fractional shortening, E = early diastolic flow velocity, A = late diastolic flow velocity, E′ = mitral annulus early diastolic velocity; Data are expressed as mean ± SEM. * *p* < 0.01 vs. normal WT and normal RasGRF1^−/−^; ^&^ *p* < 0.05 vs. diabetic WT. WT: wild type.

**Table 2:** The primer sequence.

Gene	Specific Oligonucleotide Primers for Target Sequences
Vimentin	F5′-GCAAAGATTCCACTTTGCGT-3 R5′-GAAATTGCAGGAGGAGATGC-3
Fibronectin	CGAGGTGACAGAGACCACAA-3 CTGGAGTCAAGCCAGACACA-3
NOX2	F5′-CCTCTACCAAAACCATTCGGAG-3 R5′-CTGTCCACGTACAATTCGTTCA-3
NOX4	F5′-AAGGTCCCTAGCAGGAGAACA-3 R5′-GCTACATGCACACCTGAGAAA-3
MMP-9	F5′-GCTGACTACGATAAGGACGGCA-3 R5′-TAGTGGTGCAGGCAGAGTAGGA-3
MCP-1	F5′-GCTACAAGAGGATCACCAGCAG-3 R5′-GTCTGGACCCATTCCTTCTTGG-3
CCL5	F5′-CCTGCTGCTTTGCCTACCTCTC-3 R5′-ACACACTTGGCGGTTCCTTCGA-3
Beta-actin	F-5′-CCA ACC GCG AGA AGA TGA-3 R5′-CCA GAG GCG TAC AGG GAT AG-3
